# Polyhydroxybutyrate production by recombinant *Escherichia coli* based on genes related to synthesis pathway of PHB from *Massilia* sp. UMI-21

**DOI:** 10.1186/s12934-023-02142-x

**Published:** 2023-07-14

**Authors:** Nan Jiang, Ming Wang, Linxin Song, Dengbin Yu, Shuangzi Zhou, Yu Li, Haiyan Li, Xuerong Han

**Affiliations:** 1 Engineering Research Center of Chinese Ministry of Education for Edible and Medicinal Fungi, Jilin Agricultural University, Changchun, China; 2 Jilin Province Key Laboratory of Fungal Phenomics, Jilin Agricultural University, Changchun, China; 3 School of Life Science and Technology, Changchun University of Science and Technology, Changchun, China; 4 Tianjin Institute of Industrial Biotechnology, Chinese Academy of Sciences, Tianjin, China

**Keywords:** Genome of *Massilia* sp. UMI-21, PHB metabolism-related genes, Genetically engineered bacteria, *Vgb* gene, PHB synthesis

## Abstract

**Background:**

Polyhydroxybutyrate (PHB) is currently the most common polymer produced by natural bacteria and alternative to conventional petrochemical-based plastics due to its similar material properties and biodegradability. *Massilia* sp. UMI-21, a newly found bacterium, could produce PHB from starch, maltotriose, or maltose, etc. and could serve as a candidate for seaweed-degrading bioplastic producers. However, the genes involved in PHB metabolism in *Massilia* sp. UMI-21 are still unclear.

**Results:**

In the present study, we assembled and annotated the genome of *Massilia* sp. UMI-21, identified genes related to the metabolism of PHB, and successfully constructed recombinant *Escherichia coli* harboring PHB-related genes (*phaA2*, *phaB1* and *phaC1*) of *Massilia* sp. UMI-21, which showed up to 139.41% more product. Also, the *vgb* gene (encoding *Vitreoscilla* hemoglobin) was introduced into the genetically engineered *E. coli* and gained up to 117.42% more cell dry weight, 213.30% more PHB-like production and 44.09% more product content. Fermentation products extracted from recombinant *E. coli* harboring pETDuet1-*phaA2phaB1-phaC1* and pETDuet1-*phaA2phaB1-phaC1-vgb* were identified as PHB by Fourier Transform Infrared and Proton nuclear magnetic resonance spectroscopy analysis. Furthermore, the decomposition temperature at 10% weight loss of PHB extracted from *Massilia* sp. UMI-21, recombinant *E. coli* DH5α-pETDuet1-*phaA2phaB1-phaC1* and DH5α-pETDuet1-*phaA2phaB1-phaC1-vgb* was 276.5, 278.7 and 286.3 °C, respectively, showing good thermal stability.

**Conclusions:**

Herein, we presented the whole genome information of PHB-producing *Massilia* sp. UMI-21 and constructed novel recombinant strains using key genes in PHB synthesis of strain UMI-21 and the *vgb* gene. This genetically engineered *E. coli* strain can serve as an effective novel candidate in *E. coli* cell factory for PHB production by the rapid cell growth and high PHB production.

**Supplementary Information:**

The online version contains supplementary material available at 10.1186/s12934-023-02142-x.

## Background

The heavy application of petrochemical-based plastics causes severe environmental and health issues. Polyhydroxyalkanoates (PHAs) serve as important alternative sources because of their excellent physical properties, such as similar to synthetic plastics, biodegradability and thermoplasticity [1, 2]. That kind of polymer are accumulated by numerous bacteria as carbon and energy storage substances and can be commercially produced by microbial fermentation [3]. However, the high-cost of PHA production becomes restrictions on the large-scale production and application of PHAs [4]. Therefore, exploring new and better PHA producers and genetic modification of fermentation bacteria have become important focus.

PHA-producing microbes are isolated from diverse habitats such as soil, water, waste streams and even extreme environments and about 92 bacterial genera were found can produce PHAs [5–7]. *Ralstonia eutropha* H16 is considered the model organism for PHA metabolism [8]. Also, species in *Pseudomonas*, *Azotobacter*, *Alcaligenes*, etc. are widely studied for PHA production [7, 9]. For the past few years, members in a recent descripted genus, *Massilia* [10], were reported to show a capacity of PHA accumulation [11–14]. Those *Massilia* isolates produce polyhydroxybutyrate (PHB, the most common polymer of PHAs) and have a product content from trace (lower than 1%) to about 45% of cell dry weight (Additional file 1: Table S1). Some strains from the genus *Massilia* which could grow and produce PHAs using seaweed-derived carbohydrates are considered highly promising candidates for seaweed-degrading bioplastic producers [15]. Of them, Han et al. isolated one PHA-producing microorganism from green algae *Ulva* and identified as *Massilia* sp. UMI-21, which could produce PHB from starch, maltotriose or maltose as a sole carbon source. Only polysaccharides instead of monosaccharides can be utilized by strain UMI-21, which makes it very special [14]. However, although its PHA synthase gene (*phaC*) was cloned, the genes involved in PHA metabolism in *Massilia* sp. UMI-21 are still unclear thus hindering the application.

Producing PHAs by its natural producers can be difficult since the long time and non-uniform condition to grow, accumulate and extract PHA polymers from their cells by fermentation [16]. As a widely used bacterial cell factory, *Escherichia coli* performs excellently in generating higher yields of the biopolymer because of its fast growth and easy cell lysing [17, 18]. Therefore, many PHA synthetic genes have been cloned into *E. coli* and more PHA yield and lower cost were successfully gained by this way [19, 20]. During the fermentation improving processes of genetically engineered *E. coli*, the application of *vgb* gene, encoding *Vitreoscilla* hemoglobin (VHb), plays an important role [21, 22]. VHb can efficiently bind and transport oxygen to the respiratory chain by interacting with terminal oxidase, especially under oxygen-limited conditions, showing powerful oxygen transport capacity and thus excellent performance in cell growth enhancement and PHA accumulation [23].

Here, we assembled and annotated the genome of *Massilia* sp. UMI-21 using long read sequencing, and identified genes related to the metabolism of PHB based on the genome. Then, PHB-related genes (*phaA2*, *phaB1* and *phaC1*) of strain UMI-21 were cloned and introduced into *E. coli* to produce PHB. Also, the *vgb* gene was employed in genetically engineered *E. coli* for more PHB production. The fermentation products extracted from recombinant *E. coli* were finally identified by Fourier Transform Infrared and Proton nuclear magnetic resonance spectroscopy analysis and their thermal properties were also analyzed by thermogravimetric analysis.

## Results

### Genome sequencing and identification of PHB-related genes in ***Massilia*** sp. UMI-21

**Fig. 1:**
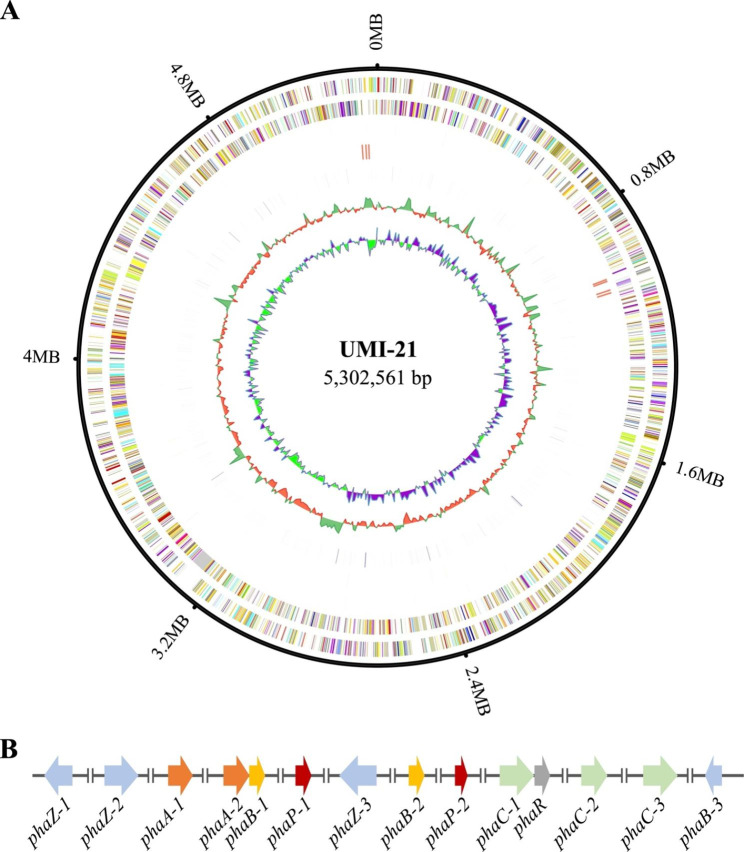
Genes related to the metabolism of PHB in *Massilia* sp. UMI-21 genome. **(A)** Circos plot of *Massilia* sp. UMI-21 genome assembly. From outside to inside: genome size, forward strand gene, reverse strand gene, forward strand ncRNA, reverse strand ncRNA, repeat units, GC content and GC-Skew. **(B)** The organization of PHB relevant genes in the genome of *Massilia* sp. UMI-21

The genome of the PHB-producing bacterium *Massilia* sp. UMI-21 was sequenced using PacBio RS II and Illumina 4000 platform, and 2871 Mb PacBio subreads and 1153 Mb Illumina data were generated respectively, resulting into a 541 × and 217 × genomic depth. The estimated genome size was 5.30 Mb with a GC content of 67.06% (Table 1; Fig. 1A). A total of 4672 genes were predicted with a total length of 4.70 Mb, accounting for 88.61% of the whole genome.

**Table 1:** General feature statistics of the *Massilia* sp. UMI-21 genome

Feature	Statistics
Genome size (bp)	5,302,561
Number of contigs	1
GC content (%)	67.06
Number of genes	4672
Total length of genes (bp)	4,698,729
Average gene length (bp)	1005.72
Gene percentage in genome (%)	88.61

To identified genes relevant to PHB metabolism in the genome of *Massilia* sp. UMI-21, Kyoto Encyclopedia of Genes and Genomes (KEGG), Clusters of Orthologous Groups (COG), NCBI Non-redundant (Nr), SwissProt, Gene Ontology (GO), TrEMBL, and EggNOG databases were used in gene function annotation. As a result, a total of 14 genes encoded for enzymes involved in PHB metabolic pathways were annotated, including 2 β-ketothiolase genes (*phaA*), 3 acetoacetyl-CoA reductase genes (*phaB*), 3 PHA synthase genes (*phaC*), 3 PHA depolymerase genes (*phaZ*), 1 PHA synthesis repressor/regulator gene (*phaR*), and 2 phasin genes (*phaP*, Table 2). To investigate the phylogenetic relationship of three important enzymes, PhaA, PhaB and PhaC, the protein sequences of the predicted 2 *phaA*, 3 *phaB* and 3 *phaC* genes in *Massilia* sp. UMI-21 and their homologs in other strains were analyzed. The phylogenetic tree suggested that PHA synthases in *Massilia* sp. UMI-21 might be Class I PhaC (Additional file 1: Fig. S1). For gene distribution, in bacterial genomes, PHA-related genes usually distribute and function in gene clusters [24]. However, those genes were scattered throughout the genome of *Massilia* sp. UMI-21. Only *phaA2* and *phaB1* constituted a *phaAB* operon, and *phaC1* and *phaR* formed a *phaCR* operon (Fig. 1B).

**Table 2:** Putative PHB-related genes in the genome of *Massilia* sp. UMI-21

Enzyme (gene)	Number	Gene ID	Size (bp)
β-ketothiolase (*phaA*)	2	UMI-21GL000143	1158
		UMI-21GL000317	1179
Acetoacetyl-CoA reductase (*phaB*)	3	UMI-21GL000318	741
		UMI-21GL001860	744
		UMI-21GL002874	597
PHA synthase (*phaC*)	3	UMI-21GL002338	1734
		UMI-21GL002806	1080
		UMI-21GL002855	1779
PHA depolymerase (*phaZ*)	3	UMI-21GL000008	1215
		UMI-21GL000085	1737
		UMI-21GL001296	1269
PHA synthesis repressor/regulator (*phaR*)	1	UMI-21GL002341	576
Phasin (*phaP*)	2	UMI-21GL000943	1779
		UMI-21GL001873	492

### Construction of genetically engineered ***Escherichia coli*** for PHB production

**Fig. 2:**
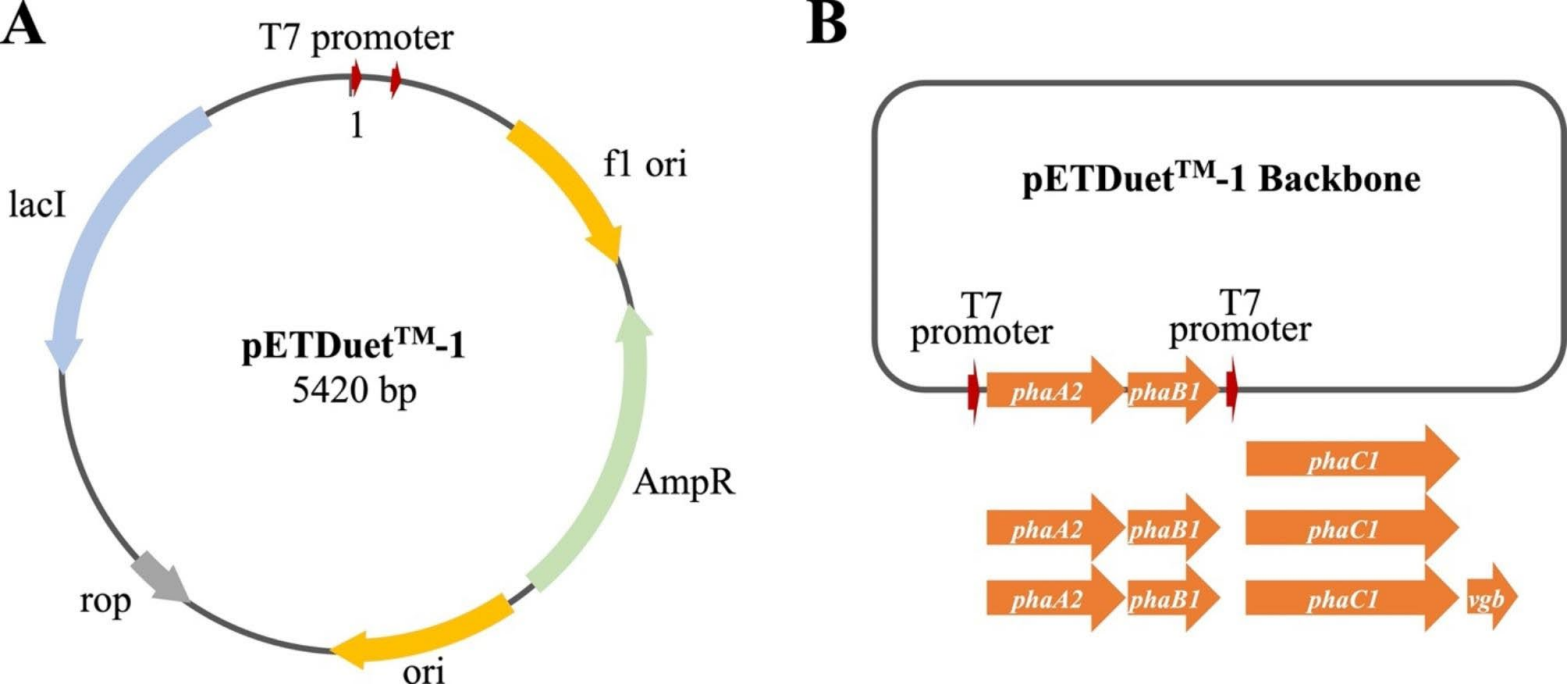
Construction process of recombinant plasmids. **(A)** Map of pETDuet1 as the plasmid backbone. **(B)** Schematic of four recombinant plasmids

Previous study has shown that the polymer produced by *Massilia* sp. UMI-21 was PHB [14]. The synthesis of PHB polymer from acetyl-CoA is sequentially catalyzed by three kinds of enzymes including β-ketothiolase, acetoacetyl-CoA reductase and PHA synthase, which are encoded by *phaA*, *phaB*, and *phaC*, respectively. We aligned the predicted 2 *phaA*, 3 *phaB* and 3 *phaC* genes from the genome of *Massilia* sp. UMI-21 to their homologous genes of the model PHA-producing strain *Ralstonia eutropha* H16 [25, 26], and found that genes UMI-21GL000317 (*phaA2*, percent identity at 75.8% in DNA and 74.9% in protein), UMI-21GL000318 (*phaB1*, percent identity at 67.7% in DNA and 60.7% in protein) and UMI-21GL002338 (*phaC1*, percent identity at 65.6% in DNA and 60.3% in protein) showed the highest sequence identity (Additional file 1: Fig. S2–S4). Also, in consideration of PHA-related genes usually cluster into operons and function in PHA synthesis [24], those three genes *phaA2*, *phaB1* and *phaC1* from *phaAB* and *phaCR* operon in *Massilia* sp. UMI-21 genome were cloned for construction of recombinant *E. coli* strains for PHB production, with the vector pETDuet1 as the plasmid backbone (Fig. 2A). First, gene fragments *phaA2phaB1* was amplified from the genome of *Massilia* sp. UMI-21, and inserted into the pETDuet1, producing pETDuet1-*phaA2phaB1*. Gene *phaC1* was then cloned into pETDuet1 and pETDuet1-*phaA2phaB1*, producing pETDuet1-*phaC1* and pETDuet1-*phaA2phaB1-phaC1*, respectively. It has been accepted that, *Vitreoscilla* hemoglobin encoded by *vgb* gene has a great help to enhance cell growth and product synthesis [21], and *vgb* gene was widely used in improving PHB accumulation [22]. Therefore, we synthesized the *vgb* gene fragment, based on the coding sequence of *vgb* gene in *Vitreoscilla* sp. C1 (NCBI accession: L21670), into recombinant plasmid pETDuet1-*phaA2phaB1-phaC1*, producing pETDuet1-*phaA2phaB1-phaC1-vgb* (Fig. 2B). Finally, genetically engineered *E. coli* DH5α harboring each plasmid of pETDuet1-*phaA2phaB1*, pETDuet1-*phaC1*, pETDuet1-*phaA2phaB1-phaC1* and pETDuet1-*phaA2phaB1-phaC1-vgb* were constructed, where the latter two were for the PHB production with the former two as controls. Results of qRT-PCR of *phaA2*, *phaB1* and *phaC1* from the two PHB-producing strains, *E. coli* DH5α-pETDuet1-*phaA2phaB1-phaC1* and DH5α-pETDuet1-*phaA2phaB1-phaC1-vgb*, validated that all three genes successfully expressed in both recombinant strains (Additional file 1: Fig. S5).

### PHB accumulation by ***E. coli*** expressing PHB-related genes of ***Massilia ***sp. UMI-21

**Fig. 3:**
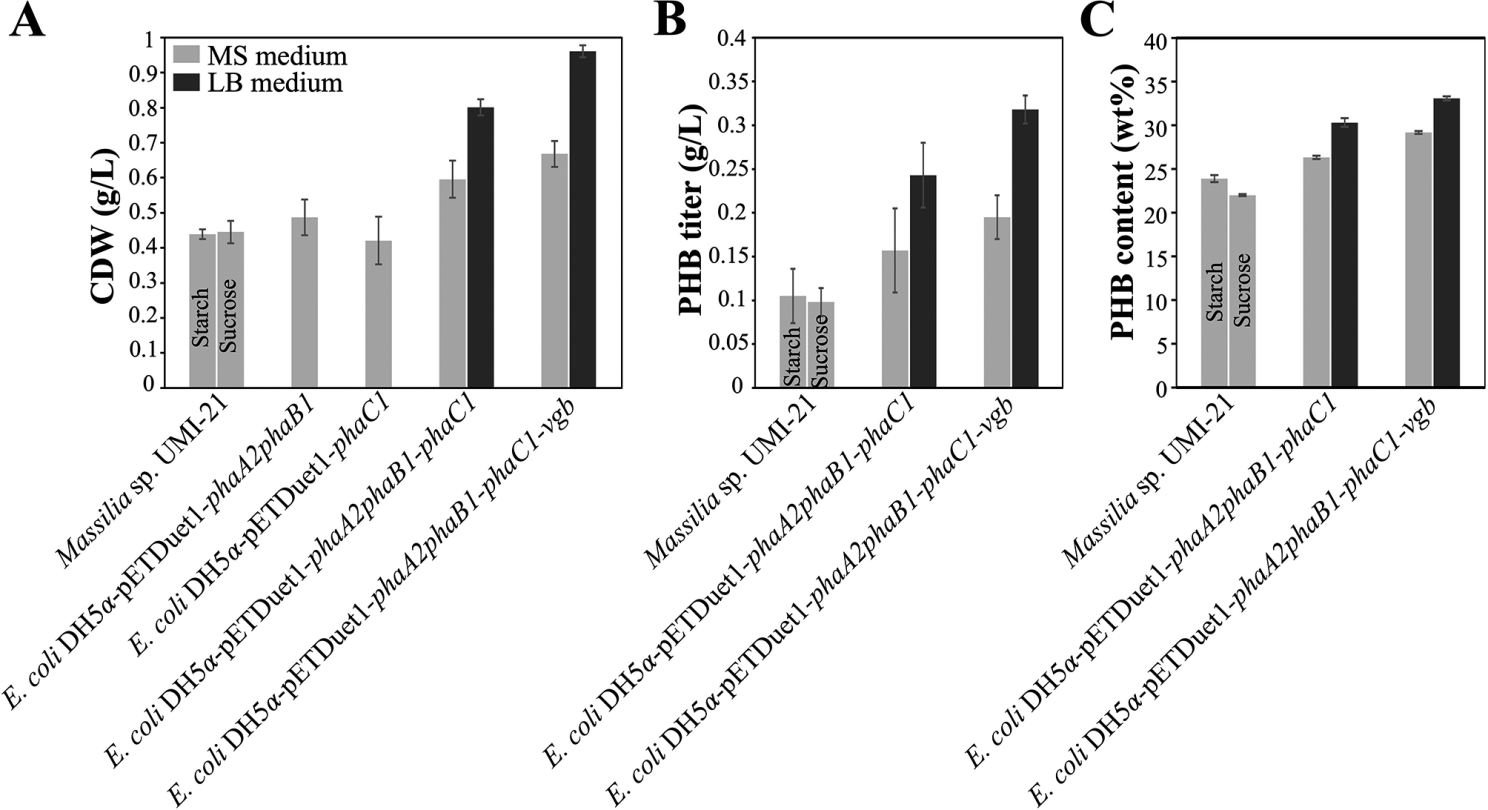
Cell growth and PHB accumulation of *Massilia* sp. UMI-21 and recombinant *E. coli* after 72 h fermentation. **(A)** Cell dry weight (CDW) of *Massilia* sp. UMI-21 and four recombinant *E. coli*. **(B)** and **(C)** PHB titer and PHB content (wt%) of *Massilia* sp. UMI-21, *E. coli* DH5α-pETDuet1-*phaA2phaB1-phaC1* and DH5α-pETDuet1-*phaA2phaB1-phaC1-vgb*

Fermentation products were extracted after 72 h shake flask experiments in Mineral Salt (MS) medium. The carbon source of recombinant *E. coli* DH5α harboring each plasmid was glucose. Since *Massilia* sp. UMI-21 could not grow when glucose was used as a carbon source [14], we employed starch and sucrose instead. For *E. coli* DH5α-pETDuet1-*phaA2phaB1-phaC1* and DH5α-pETDuet1-*phaA2phaB1phaC1-vgb*, Luria-Bertani (LB) medium supplemented with glucose was also used to investigate the influence of different medium (Fig. 3).

The cell dry weight (CDW) of *Massilia* sp.UMI-21 with starch and sucrose as carbon sources had comparable results of 0.439 g/L and 0.445 g/L, respectively. Similarly, genetically engineered *E. coli* DH5α-pETDuet1-*phaA2phaB1* and DH5α-pETDuet1-*phaC1* could also grow on the glucose contained MS medium with a 0.487 g/L and 0.421 g/L CDW. In the MS medium, recombinant *E. coli* DH5α harboring pETDuet1-*phaA2phaB1-phaC1* and pETDuet1-*phaA2phaB1-phaC1-vgb* had the highest CDW with 0.596 g/L and 0.668 g/L, increasing by 34.84% and 51.13% than strain UMI-21, respectively. When cultured in LB medium, those two recombinant strains showed higher CDW, up to 0.801 g/L and 0.961 g/L with approximately 81.22% and 117.42% increase (Fig. 3A).

As expected, no PHA was extracted from *E. coli* DH5α-pETDuet1-*phaA2phaB1* and DH5α-pETDuet1-*phaC1* since lack of complete set of genes for PHB synthesis. The PHA titer and content (wt%) of recombinant *E. coli* DH5α-pETDuet1-*phaA2phaB1-phaC1* and DH5α-pETDuet1-*phaA2phaB1-phaC1-vgb* in MS medium was 0.157 g/L (26.34%) and 0.195 g/L (29.19%) respectively, which were higher than *Massilia* sp. UMI-21, 0.105 g/L (23.91%) and 0.098 g/L (22.02%) with starch and sucrose as carbon sources. That means about 54.68% and 92.12% increase in product titer, and 14.70% and 27.11% increase in product content. In LB medium, the performance of the two PHB-producing genetically engineered *E. coli* were even better, with 0.243 g/L and 0.318 g/L product titer and 30.33% and 33.09% PHB content. Compared with *Massilia* sp. UMI-21, PHB production increased 139.41% and 213.30%, and PHB content increased 32.07% and 44.09% (Fig. 3B, C).

### Characterization of fermentation products

**Fig. 4:**
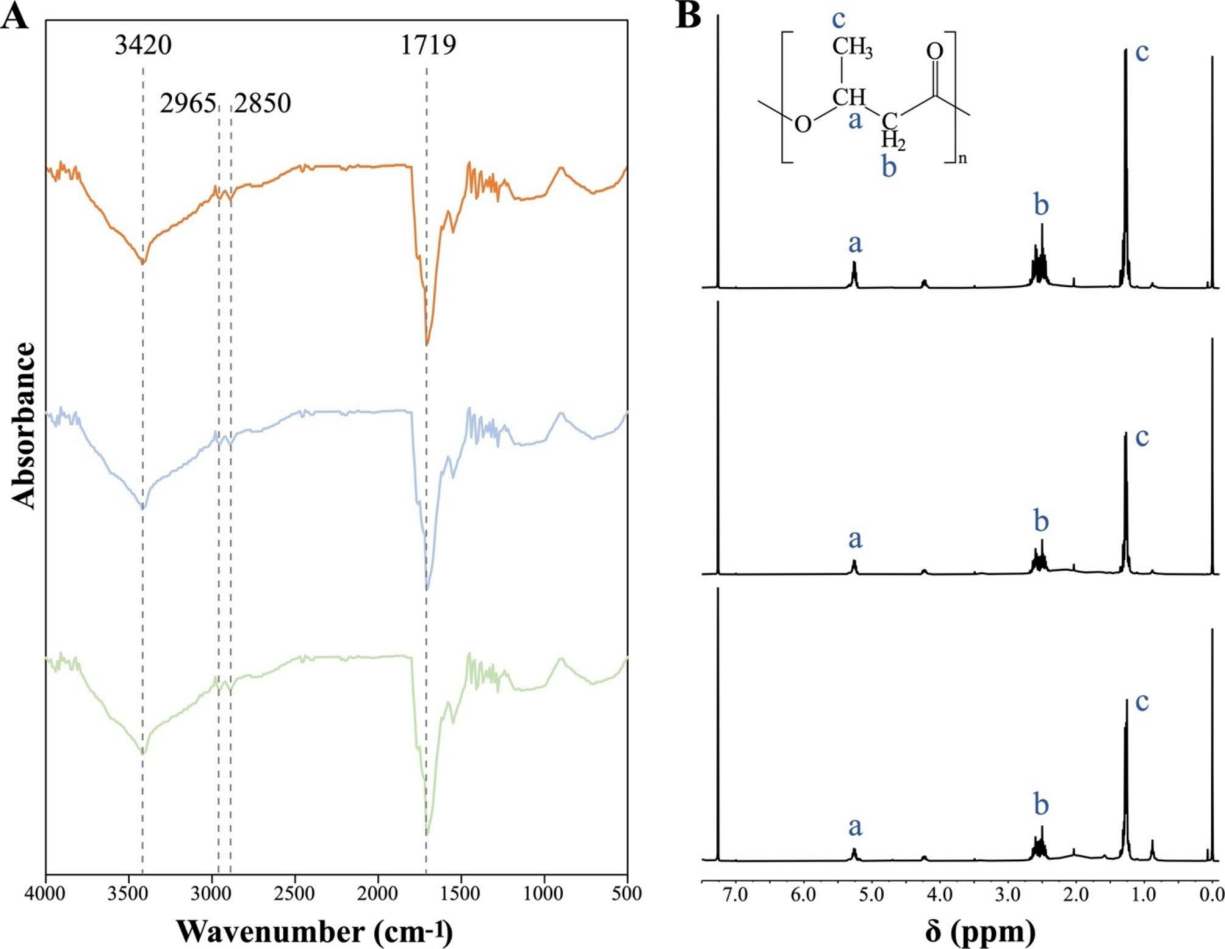
Characterization of fermentation products. **(A)** Results of FT-IR analysis. (**B)** Results of ^1^H-NMR analysis. Plots from top to bottom show results of *Massilia* sp. UMI-21, *E. coli* DH5α-pETDuet1-*phaA2phaB1-phaC1* and DH5α-pETDuet1-*phaA2phaB1-phaC1-vgb*, respectively

In order to identify structure and determine properties of fermentation products by genetically engineered *E. coli*, Fourier Transform Infrared spectroscopy (FT-IR), proton nuclear magnetic resonance (^1^H-NMR), and thermogravimetric analysis (TGA) were conducted. Fermentation products in MS medium from recombinant *E. coli* DH5α, harboring plasmid pETDuet1-*phaA2phaB1-phaC1* and pETDuet1-*phaA2phaB1-phaC1-vgb*, and *Massilia* sp. UMI-21 with glucose and sucrose as the carbon source were used in this study. Consistent with the characteristic absorption bands of PHB products produced by *Massilia* sp. UMI-21, the FT-IR results of *E. coli* DH5α-pETDuet1-*phaA2phaB1-phaC1* and DH5α-pETDuet1-*phaA2phaB1-phaC1-vgb* products showed absorption bands at 3420 cm^− 1^ (-OH), 1719 cm^− 1^ (C = O), and 2850–2965 cm^− 1^ (C-H), indicating the fermentation products of recombinant *E. coli* was PHB (Fig. 4A). Also, the results of ^1^H-NMR analysis suggested that the polymer produced by recombinant *E. coli* was PHB, same with the fermentation products from strain UMI-21 (Fig. 4B).

**Fig. 5:**
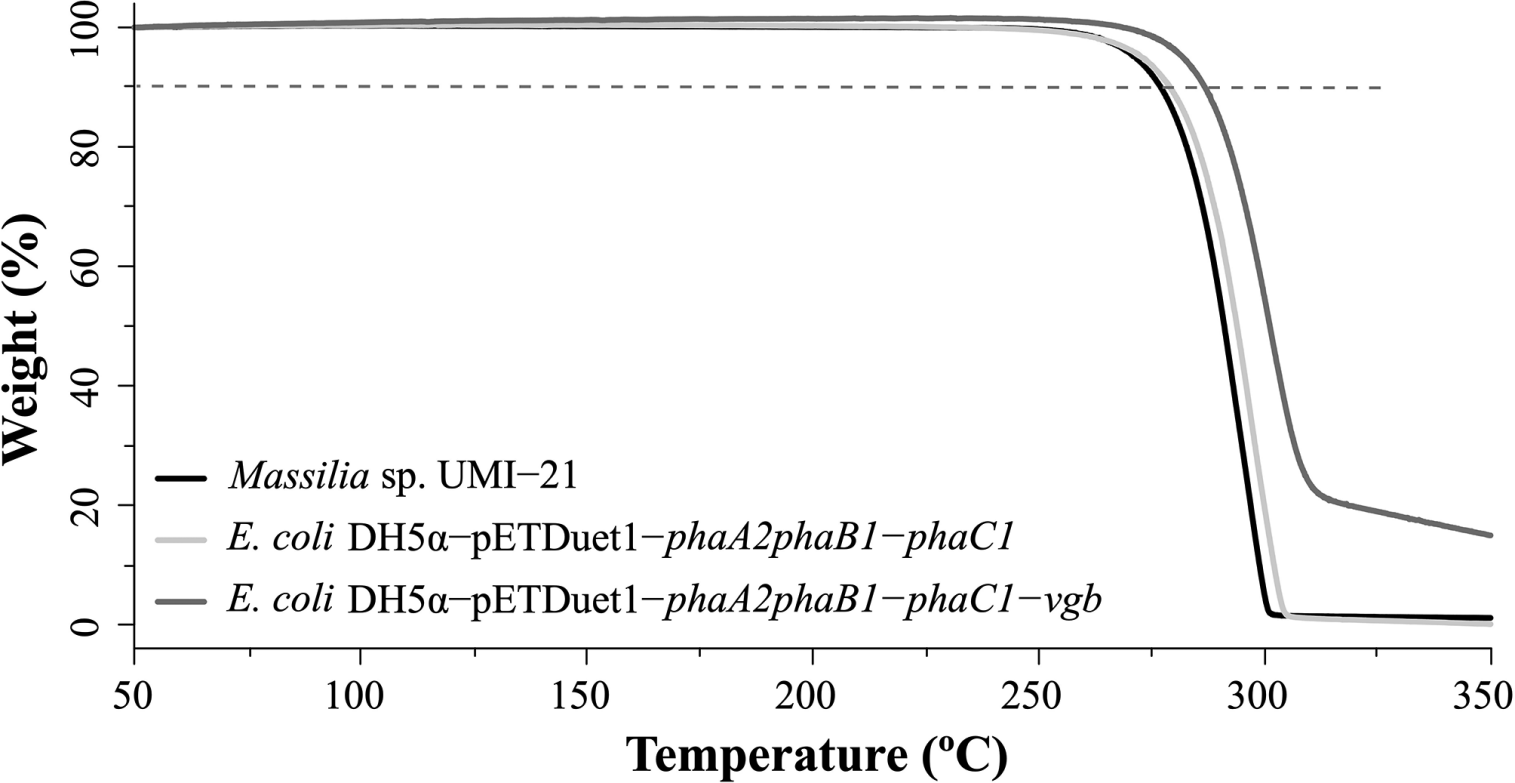
TGA of fermentation products from *Massilia* sp. UMI-21 and recombinant *E. coli* harboring pETDuet1-*phaA2phaB1-phaC1* and pETDuet1-*phaA2phaB1-phaC1-vgb*

Thermal stability of PHAs is important for their melt processing. The results of TGA showed that the initial decomposition temperature (T10%, temperature at 10% weight loss) of PHB extracted from recombinant *E. coli* DH5α-pETDuet1-*phaA2phaB1-phaC1* and DH5α-pETDuet1-*phaA2phaB1-phaC1-vgb* was 278.7 and 286.3 °C, respectively, which was in approximately the same T10% of PHB produced by *Massilia* sp. UMI-21 with 276.5 °C (Fig. 5).

## Discussion

Researches focusing on screening and construction of excellent, new and genetically engineered PHA-producing strains provide a theoretical basis for large-scale popularization and application of PHAs. In this study, we assembled and annotated the genome of *Massilia* sp. UMI-21, a PHB-producing bacterium isolated from previous study [14], and successfully constructed recombinant *E. coli* harboring PHB-related genes (*phaA2*, *phaB1* and *phaC1*) of strain UMI-21, which showed more PHB accumulation than *Massilia* sp. UMI-21. Also, the *vgb* gene was introduced into the genetically engineered *E. coli* and the PHB production was further increased.

We identified 14 genes encoded for enzymes involved in PHB metabolic pathways in the genome of *Massilia* sp. UMI-21, including 2 *phaA*, 3 *phaB*, 3 *phaC*, 3 *phaZ*, 1 *phaR*, and 2 *phaP* (Table 2). With those clues, we can reasonably conclude that in *Massilia* sp. UMI-21, two Acetyl Coenzyme A molecules condense into one acetoacetyl-CoA molecule with the help of β-ketothiolase (PhaA), then acetoacetyl-CoA reductase (PhaB) reduces Acetoacetyl-CoA to 3-hydroxybutyryl-CoA (3HB-CoA), and finally 3HB-CoA is polymerized into PHB by PHA synthase PhaC [25, 26]. Since the discovery of *phaCAB* operon in model PHA-producing *R. eutropha* H16, genes relevant to PHA metabolism were usually found clustered together in bacterial genome [26–29]. Interestingly, in the genome of *Massilia* sp. UMI-21, PHA-related genes were disjointed except for *phaA2B1* and *phaC1R* (Fig. 1B). That scattered distribution pattern was also found in the genome of other PHA-producing bacteria, such as *Neptunomonas concharum* JCM17730, which could accumulate PHB using fructose as the carbon source [30, 31]. However, further investigations are still needed to provide insights into whether and how gene distribution affects PHA synthesis.

**Fig. 6:**
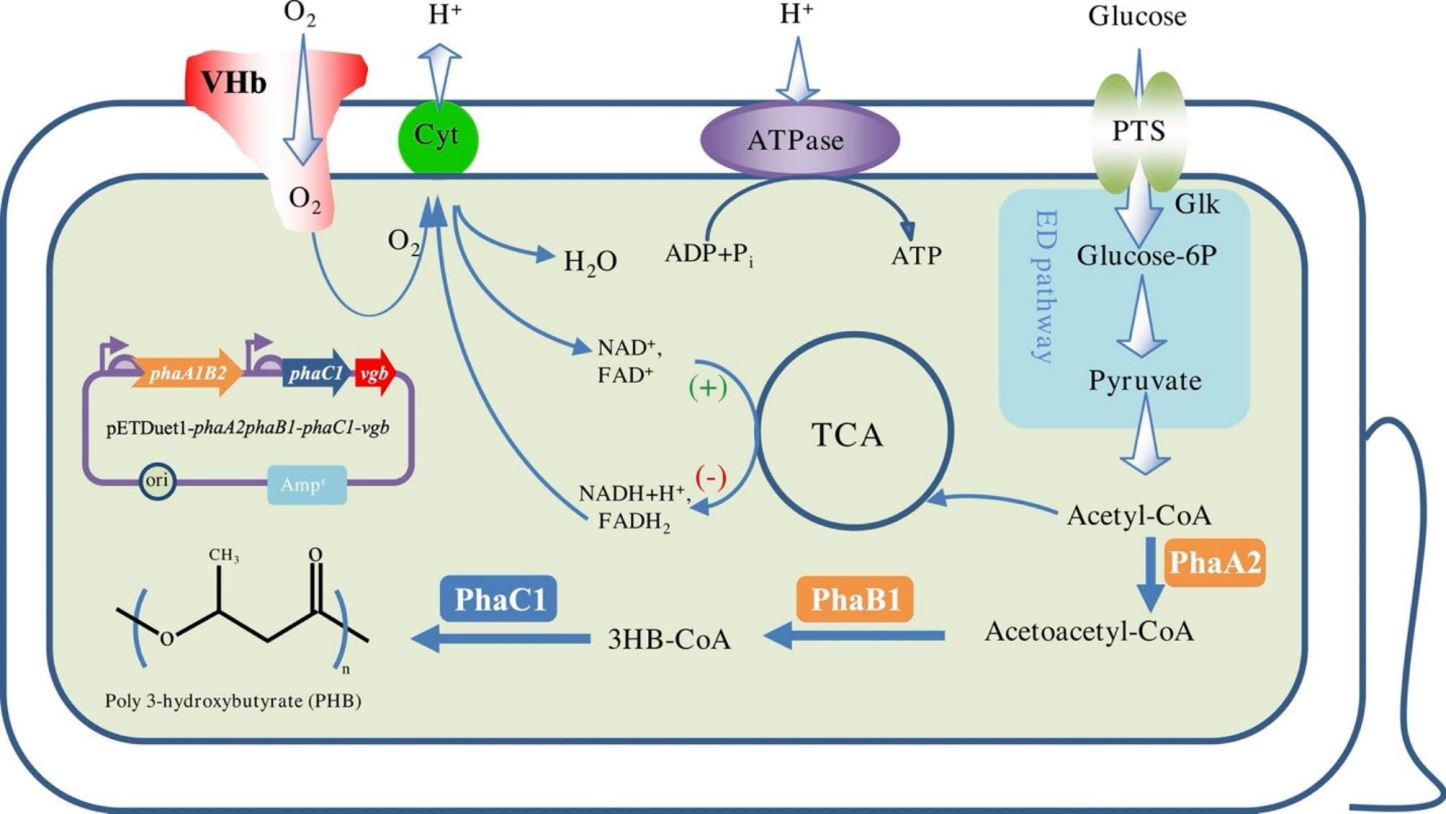
Putative PHB metabolism pathway in *E. coli* DH5α-pETDuet1-*phaA2phaB1-phaC1-vgb*. (+) in green denotes activation; (-) in red denotes repression; Glucose-6P: glucose-6-phosphate; Glk: glucokinase; PTS: phosphotransferase system; Cyt: cytochrome; TCA: tricarboxylic acid cycle; VHb: *Vitreoscilla* hemoglobin

Heterologous expression of *phaA2*, *phaB1* and *phaC1* in *E. coli* resulted in PHB accumulation, showing that those three genes are crucial in the PHB metabolic pathway in *Massilia* sp. UMI-21. The putative PHB metabolism pathway in recombinant *E. coli* DH5α-pETDuet1-*phaA2phaB1-phaC1-vgb* was shown in Fig. 6, where VHb encoded by *vgb* gene enhances aerobic respiration and consequently increases the consumption of NADH and generation of NAD^+^, which contributes to PHB synthesis under the cooperation of PhaA2, PhaB1 and PhaC1. *E. coli* as a widely used engineered bacterium has more advantages than other bacteria in PHB production. For example, mature and versatile *E. coli* cultivation techniques enable mass production [32], PHA production in *E. coli* can be carried out using a wide range of carbon sources making it possible to reduce the cost of raw materials [33], and since the absence of depolymerase in the cell of *E. coli*, PHA polymers do not degrade during fermentation resulting in more product accumulation [34]. In this work, we obtained 54.68–139.41% more PHB accumulation than in *Massilia* sp. UMI-21 in cultivation of recombinant *E. coli* harboring pETDuet1-*phaA2phaB1-phaC1* in MS or LB medium. Compared with similar work from de Almeida et al. (2010), in which they produced PHB using recombinant *E. coli* carrying *phaBAC* of *Azotobacter* sp. FA8 and obtained 20.4% PHB content in MYA medium with glucose, our work got higher production in both MS and LB medium with the same carbon source [35], showing PHB-related genes in *Massilia* sp. UMI-21 has the potential for applications. In addition, the further improvement, namely the introduction of *vgb* gene, resulted into 92.12% (in MS medium) and 213.30% (in LB medium) increase of PHB production (Fig. 3). Similar important roles of *vgb* gene for improving microbial fermentation processes have been widely observed [22]. For example, Horng et al. (2010) overexpressed the artificial *phaCAB-vgb* operon in *E. coli* and got 23%, 57% and 93% more CDW, PHB content and PHB concentration, respectively [36]. Products extracted from *E. coli* DH5α-pETDuet1-*phaA2phaB1-phaC1* and DH5α-pETDuet1-*phaA2phaB1-phaC1-vgb* was identified as PHB by FT-IR and ^1^H-NMR analysis (Fig. 4), which was identical to that from *Massilia* sp. UMI-21. Also, the thermal properties of PHB from our recombinant *E. coli* and strain UMI-21 were resembled with an approximately 280 °C initial decomposition temperature (Fig. 5), similar with the standard PHB (T10% ~270–290 °C) and about 20–65 °C higher than PHB extract from other organism, such as *Burkholderia sacchari* (T10% ~ 263 °C), *Bacillus* sp. CYR1 (T10% ~ 230 °C) and *Micrococcus luteus* (T10% ~ 224 °C), showing better thermal stability [9, 37, 38].

## Conclusions

In this work, we presented genome assembly of PHB-producing *Massilia* sp. UMI-21 and further constructed recombinant *E. coli* based on genes related to synthesis pathway of PHB mined from the genome for PHB production. The results that PHB successfully and more accumulated in the recombinant *E. coli* harboring *phaA*, *phaB* and *phaC* showed these three might be key genes for PHB synthesis in strain UMI-21. Also, we introduced the *vgb* gene into recombinant *E. coli* and gained more PHB production and the fermentation products showed good properties. PHB synthesis by recombinant *E. coli* using monosaccharides is more efficient than that by *Massilia* sp. UMI-21 using starch, which could reduce the cost of PHB production. Thus, the genetically engineered *E. coli* DH5α-pETDuet1-*phaA2phaB1-phaC1-vgb* constructed in the present study can serve as an effective new strain for PHB synthesis by its rapid cell growth and high PHB production. Although further examination is needed to realize large scale PHB production, during the improving process such as fermentation optimization, this novel recombinant strain could contribute to the industrial low-cost PHA production. Moreover, the association study of other PHA-related genes will provide molecular basis for future synthetic biology research in novel PHAs.

## Methods

### Genome sequencing, assembly and annotation

Bacterial cells of *Massilia* sp. UMI-21 from overnight cultures were used for *de novo* genome sequencing with PacBio RS II and Illumina HiSeq 4000 platform at the Beijing Genomics Institute (BGI, Shenzhen, China). Genomic DNA was extracted using the FastPure Bacteria DNA Isolation Mini Kit (Vazyme, Nanjing, China). Draft genome was assembled using the Celera Assembler against a high-quality corrected circular consensus sequence subreads set [39]. To improve the accuracy of the genome sequences, GATK and SOAP tool packages (SOAP2, SOAPsnp, SOAPindel) were used to make single-base corrections [40, 41]. To trace the presence of any plasmid, the filtered Illumina reads were mapped using SOAP to the bacterial plasmid database (http://www.ebi.ac.uk/genomes/plasmid.html, last accessed July 8, 2016). Finally, the genome assembly of *Massilia* sp. UMI-21 was submitted to GenBank under the BioProject PRJNA669114.

For gene prediction, Glimmer3 with a Hidden Markov Model was employed [42]. Non-coding RNA, including tRNA, rRNA and sRNA was recognized with tRNAscan-SE, RNAmmer, and Rfam database [43–45]. The tandem repeats annotation was obtained using the Tandem Repeat Finder (http://tandem.bu.edu/trf/trf.html), and the minisatellite DNA and microsatellite DNA selected based on the number and length of repeat units. For gene function annotation, the best hit abstracted using BLAST alignment tool based on seven databases (Kyoto Encyclopedia of Genes and Genomes (KEGG), Clusters of Orthologous Groups (COG), NCBI Non-redundant (Nr), SwissProt, Gene Ontology (GO), TrEMBL, and EggNOG database) was used [46–52]. Sequence alignment was performed using Clustal Omega (https://www.ebi.ac.uk/Tools/msa/clustalo/) and pairwise sequence alignment (https://www.ebi.ac.uk/Tools/psa/) tools available at EMBL-EBI for multiple and pairwise sequence alignment, respectively. Phylogenetic trees were displayed by the online tool of iTOL v5 (https://itol.embl.de).

### Construction of genetically engineered ***Escherichia coli***

Molecular cloning and manipulation of plasmids were done with *E. coli* DH5α. For construction of recombinant plasmid, the vector pETDuet1 was used as a backbone. Gene fragments were amplified using KOD-Plus-Neo DNA polymerase (TOYOBO, Osaka, Japan) and primers listed in Additional file 1: Table S2, which were synthesized by Jilin KuMei company (Changchun, China). Restriction enzymes used for vector linearization were purchased from the New England Biolabs (Beijing) LTD (Additional file 1: Table S2). All seamless cloning between gene fragments and linear vectors was using the ClonExpress Ultra One Step Cloning Kit (Vazyme, Nanjing, China). First, PHB-related genes *phaA2B1* was amplified from the genome of *Massilia* sp. UMI-21 and seamless cloned into pETDuet1 vector, which resulted in pETDuet1-*phaA2phaB1*. Second, gene *phaC1* was inserted into pETDuet1 and pETDuet1-*phaA2phaB1*, producing pETDuet1-*phaC1* and pETDuet1-*phaA2phaB1-phaC1*. The same method was used in the construction of recombinant plasmid pETDuet1-*phaA2phaB1-phaC1-vgb*. Based on the coding sequence of *vgb* gene from *Vitreoscilla* sp. C1 in NCBI (accession L21670), the *vgb* gene fragment was synthesized and connected to the pUC57 vector by Jilin KuMei company (Changchun, China). Then, the *vgb* gene fragment was amplified using the pUC57-*vgb* plasmid as template and seamless cloned into pETDuet1-*phaA2phaB1-phaC1* (Additional file 1: Table S2). Finally, pETDuet1-*phaA2phaB1*, pETDuet1-*phaC1*, pETDuet1-*phaA2phaB1-phaC1* and pETDuet1-*phaA2phaB1-phaC1-vgb* was transformed into *E. coli* DH5α using the heat shock method, respectively.

### Cultivation of genetically engineered ***E. coli*** and extraction of products

PHB accumulation were carried out in *Massilia* sp. UMI-21 and four genetically engineered *E. coli* DH5α harboring each plasmid. For strain UMI-21, cells were activated in R2A liquid medium (Hopebio, Qingdao, China) at 30 °C, 150 rpm for 24 h followed by a 24 h pre-culture with a 5% (v/v) inoculation in the same condition. A total of 5% (v/v) pre-culture was then inoculated into nitrogen-limiting Mineral Salt (MS) medium with 1% (w/v) cold water-soluble starch or sucrose and incubated at 30 °C, 150 rpm for 72 h (all reagents without explicitly stated were purchased from Beijing Chemical Plant and of analytical grade) [14]. For recombinant strains, cell activation and pre-culture were carried out in Luria-Bertani (LB) liquid medium at 37 °C, 180 rpm for 12 and 24 h, respectively. Then, 5% (v/v) pre-culture was inoculated into nitrogen-limiting MS with 1% (w/v) glucose as a carbon source and incubated at 37 °C, 180 rpm for 72 h. LB medium supplemented with 1% (w/v) glucose were also used for PHB accumulation in *E. coli* DH5α-pETDuet1-*phaA2phaB1-phaC1* and DH5α-pETDuet1-*phaA2phaB1phaC1-vgb*.

After 72 h shake flask fermentation, the culture was harvested by centrifugation at 4 °C, 10,000 rpm for 10 min and washed twice with deionized water. The bacteria were rapid frozen at -80 °C and finally lyophilized to constant weight by FD-1B-80 vacuum freeze dryer (Biocool, Beijing, China). The cell dry weight (CDW) was used to assess the growth of strains.

PHB was extracted from the bacterial cell by the chloroform/methanol method. The lyophilized bacteria were ground into powder, mixed with chloroform and ultrasonic crushed for 10 min followed by shaking at 30 °C, 110 rpm for 48 h. After suction filtration and rotatory evaporation, prechilled methanol (five times the volume) was mixed in and placed at 4 °C overnight for precipitation. PHB was gained after a second suction filtration and drying. PHB production was determined by the dry weight of extracted PHB. The PHB content was defined as the percent ratio of PHB production to CDW.

### Fourier Transform Infrared spectroscopy (FT-IR) analysis

A total of 2 mg dry products extracted from *Massilia* sp. UMI-21, *E. coli* DH5α-pETDuet1-*phaA2phaB1-phaC1* and DH5α-pETDuet1-*phaA2phaB1phaC1-vgb* were mixed with potassium bromide (KBr) in 1:50 ratio to form samples and qualitative characterized by FT-IR analysis, which was carried out in IRAffinity-1 S instrument (SHIMADZU, Japan) for 15 scans and a resolution of 1 cm^− 1^.

### Proton nuclear magnetic resonance (^1^H-NMR) spectroscopy

The ^1^H-NMR spectroscopy was also used to identify the structure of fermentation products. Thoroughly dissolved in deuterated chloroform (CDCl_3_), 5 mg polymers were used to obtain the ^1^H-NMR spectra using the AVANCE NEO (400 MHz) spectrometer (Bruker, Germany) at 90 °C with a 4 ms, 3,000 Hz spectral width and a 4 s repetition rate.

### Thermogravimetric analysis (TGA)

TGA was performed to determine the thermal stability in terms of weight loss of extracted polymer as a function of temperature. A total of 10 mg samples were analyzed by STA449C thermal analyzer (Netzsch, Germany) using a scanning rate of 10 °C/min with temperature increasing from 50 to 350 °C under nitrogen.

## Electronic supplementary material

Below is the link to the electronic supplementary material.

**Additional file 1**: Table S1. PHB-producing strains in the genus *Massilia*. Table S2. Primers used in recombinant plasmid construction. Fig. S1. Phylogenetic tree of PHA-related proteins from *Massilia* sp. UMI-21 and related taxa. (A) PhaA. (B) PhaB. (C) PhaC. The GenBank accession numbers were shown in the parentheses. Green denotes Class III and IV PhaC; orange denotes Class I and II PhaC. Fig. S2. Results of *phaA* gene and amino acid (AA) sequence alignment between *Ralstonia eutropha* H16 and *Massilia* sp. UMI-21. (A) Alignment of DNA sequences. (B) Alignment of AA sequences. Fig. S3. Results of *phaB* gene and amino acid (AA) sequence alignment between *Ralstonia eutropha* H16 and *Massilia* sp. UMI-21. (A) Alignment of DNA sequences. (B) Alignment of AA sequences. Fig. S4. Results of *phaC* gene and amino acid (AA) sequence alignment between *Ralstonia eutropha* H16 and *Massilia* sp. UMI-21. (A) Alignment of DNA sequences. (B) Alignment of AA sequences. Fig. S5. Relative expression of *phaA2* (A), *phaB1* (B) and *phaC1* (C) genes in wild *E. coli* DH5α strain and the recombinant *E. coli* DH5α-pETDuet1-*phaA2phaB1-phaC1* and DH5α-pETDuet1-*phaA2phaB1-phaC1-vgb* strains by qRT-PCR. 16S rRNA serves as the internal control

## Data Availability

Genome data of *Massilia* sp. UMI-21 were submitted to GenBank (https://www.ncbi.nlm.nih.gov/genbank/) under the BioProject PRJNA669114.
